# Construction Notes

**Published:** 1894-03-24

**Authors:** 


### CONSTRUCTION NOTES.

#### Extension of Keighley Cottage Hospital.

The work of enlarging the Keighley Cottage Hospital is on the point of completion, so that shortly, instead of an institution with barely room for ten beds, the district will have ready for its needs accommodation for five times that number. The word "Cottage" is an accretion to the original title, and will probably be dropped. At present it is thought that four wards will be furnished for adults and one for children. Messrs. W. and J. Bailey, architects, submitted the plans for this extension, involving an expenditure of £1,091. Beyond the accepted contracts extras have been passed, including an additional length to the south of ten feet, a permanent mortuary and washhouse, a hoist of the latest design from the operating theatre to the upper floor, furnishing throughout, &c. The whole requires an outlay probably not far short of £2,000. There is about £1,500 to meet the new liabilities, so that the balance of £500 will need to be raised before the institution can be said to be on a sound footing.

